# Supplementary material for: Acceptance and compliance with micronutrient powder (MNP) among children aged 6–23 months in northern Nigeria
Source: PLOS Glob Public Health. 2022 Oct 17;2(10):e0000961. doi: 10.1371/journal.pgph.0000961 (PMC10022258; doi:10.1371/journal.pgph.0000961)
Supplement: S1 Dataset — (ZIP) [file pgph.0000961.s008.zip › S8_Dataset/Direct Observations.zip!/Direct Observations/05072015_A_SO_AS_2_DO.pdf]

1.1.1 Instrument F –Direct Observation Form

<<Instrument F Direct Observation Form>>

PI Name: PragyaMathema,Stephen Kodish
Study Title: Formative research to develop an infant and young child feeding (IYCF) intervention with micronutrient powder (MNP) for children aged 6 – 23 months
PI Version/Date: Version1.0/January 24, 2015

Date: _July 05, 2015_____________Location: _Bongo community, LGA -Song

Data Collector: Antiya Sanda Participant ID: A house by the road side

Main goals of this direct observation are to answer the following questions:

1. In this household, what would be the best way(s) to give nutritional supplements to young children? Why?

2. In this household, describe the food sharing that you observe throughout the meal. Are there any predictable patterns?

3. In this household, who is favored and how is this favoritism expressed during meal preparation and eating?

(TEAR OFF AT PERFORATED DOTTED LINE BELOW)

PARTICIPANT ID: __

Household Composition Table (Fill out as you do data collection)

INITIALS	SEX (M/F)	AGE (estimated)	Relationship to household	Description
FB	F	31 YRS	Mother	A fat black woman.
SB	F	22 MONTHS	Child (observed child)	A small black baby.
ST	M	35 Yrs	Father	A slim tall man wearing a green shirt
FS	F	4 Yrs	daughter	A fair slim female child
TS	F	8 Yrs	Daughter	A tall slim girl

PARTICIPANT ID: __________

TIME	ACTOR INITIALS	RECIPIENT INITIALS	WHAT HAPPENED	FOOD	Reactivity
8.00	FB		Fetching water		
8:05	FB		Fetching water from the water pot		
8:10	FB		Carry firewood		
8:15	FB		Sitting on the mat		
8:20	FB		Bring dirty plats from the kitchen		
8:25	FB		Gathers all the dirty plates together in a big bowl		
8:30	FB		Start fetching water in a big bucket		
8:35	FB		Start washing the plates		
8:40	FB		Washing the plates		
8:45	FB		Washing the plates		
8:50	FB		Washing the plates		
8:55	FB		Washing the plates		
9:00	FB		Start making fire		
9:05	FB		Making fire		
9:10	FB		Fetches water in a pot		
9:15	FB		Puts pot on the fire		
9:20	FB		Covers the pot		
9:25	FB		Bring guinea corn	Guinea corn	
9:30	FB		Pour guinea corn flour into the cup	Guinea corn	

TIME	ACTOR INITIALS	RECIPIENT INITIALS	WHAT HAPPENED	FOOD	Reactivity
9:35	FB		Pour little water into the guinea corn flour		
9:40	FB		Start mixing guinea corn flour	Guinea corn and water	
9:45	FB		Opens the pot on the fire and poured the mixed paste into it	Guinea corn	
9:50	FB		Use a stick to stir the mixture on the fire		
9:55	FB		Adds groundnut paste into the mixture on fire	Guinea corn and groundnut paste	
10:00	SB		Start crying		
10:05	FB		Blowing the fire		
10:10	FB	SB	Carry her brother on her arms		
10:15	FB		Making kunu		
10:20	FB		Bring down the pot from the fire	Guinea corn	
10:25	FB		Remove the mixing stick		
10:30	FB		Bring two cups		
10:35	FB		Pours kunu into the two cups	Guinea corn	
10:40	FB		Pouring kunu into the cups		
10:45	TS		Bring her cup		
10:50	FB		Shares all the kunu into three cups	Kunu dawa	
10:55	FB		Removes all the firewood		
11:00	SB		Comes to carry his cup of kunu	Guinea corn	
11:05	FB	SB	Carry the baby and cup of kunu		
11:10	FS, TS		Comes and pick their cups of kunu		
11:15	FB	SB	Goes into the room while carrying her son		
11:20	FB	SB	Comes out of the room with 1 sachet of MNP		
11:25	FB	SB	Keep her child on the mat		

TIME	ACTOR INITIALS	RECIPIENT INITIALS	WHAT HAPPENED	FOOD	Reactivity
11:30	FB		Start unsealing MNP		
11:30	FB		Taste the kunu to know if it is cold	Kunu dawa	
11:35	FB		Pour MNP into the kunu	MNP, Kunu dawa	
11:40	FB		Stirs the MNP and kunu together	Kunu dawa, MNP	
11:45	FB		Serving kunu from flask into a cup.	Kunun	
11:55	FB		Use a spoon to taste it	Kunun dawa	
12:00	FB	SB	Carry her child on the lap		
12:05	FB	SB	Start feeding the child with MNP mixed kunu	Kunu dawa	
12:10	FB	SB	Feeding her child	Kunu dawa	
12:15	FB	SB	Feeding her child	Kunu dawa	
12:20	FB	SB	Feeding her child	Kunu dawa	
12:25	FB	SB	Feeding her child	Kunu dawa	
12:30	FB	SB	Feeding her child	Kunu dawa	
12:35	FB	SB	Feeding her child	Kunu dawa	
12:40	FB	SB	Feeding her child	Kunu dawa	
12:45	SB		Start feeding himself with the small quantity of kunu left		
12:50	SB		Finish the kunu and keep the empty cup on the ground		
1:00	SB		Start playing with the empty cup of kunu		
1:05	SB		Lying on the mat		
1:10	FB		Collect the empty cup to rinse it		
1:15	FB		Fetches water in a cup		

TIME	ACTOR INITIALS	RECIPIENT INITIALS	WHAT HAPPENED	FOOD	Reactivity
1:20	FB		Uses the water in the cup to wash the face of her child		
1:25	FB		Picks a broom		
1:30	FB		Start sweeping		
1:35	SB		Playing on the mat		
1:40	FB		Sweeping		
1:45	FB		Sweeping		
1:50	FB		Sweeping the compound		
1:55	FB		Sweeping the compound		
2:00	FB		Chops the broom		
2:05	SB		Start sleeping		
2:10	SB		Sleeping		
2:15	SB		Sleeping		
2:20	SB		Sleeping		
2:25	SB		Sleeping		
2:30	SB		Sleeping		
2:35	SB		Sleeping		
2:40	SB		Sleeping		
2:45	SB		Sleeping		
2:50	SB		Sleeping		
2:55	FB		Sitting next to her child		
3:00	TS	FB	Gives her mum the leaf	Spinach	
3:10	FB		Collect the leaf		
3:15	TS		Bring a big bowl to the mom		

TIME	ACTOR INITIALS	RECIPIENT INITIALS	WHAT HAPPENED	FOOD	Reactivity
3:20	TS		Bring knife also		
3:25	FB		Start picking the leaves	Spinach	
3:30	FB		Picking the green leaves	Spinach	
3:35	FB		Picking the green leaves	Spinach	
3:40	FB		Picking the green leaves	spinach	
3:45	FB		Picking the green leaves	spinach	
3:50	FB		Picking the green leaves	spinach	
3:55	FB		Picking the green leaves	spinach	
4:00	FB		Cutting the green leaves with knife	spinach	
4:05	FB		Cutting the green leaves with knife	spinach	
4:10	FB		Cutting the green leaves with knife	spinach	
4:15	FB		Cutting the green leaves with knife	spinach	
4:20	FB		Cutting the green leaves with knife	spinach	
4:25	FB		Goes into the kitchen		
4:30	FB		Brings tuwo in a plate with soup	Maize meal and okro	
4:35	FB		Calls all her children to eat		
4:40	SB,FS		Eating together	Maize meal and okro soup	
4:45	SB,FS		Eating together tuwo masara	Maize meal and okro soup	
4:50	SB,FS		Eating together	Maize meal and okro soup	
4:55	Ts		Comes in and join them to eat	Maize an okro soup	
5:00	SB,FS,		Eating together and playing	Maize	

TIME	ACTOR INITIALS	RECIPIENT INITIALS	WHAT HAPPENED	FOOD	Reactivity
	TS			and okro	
5:05	FS	SB	Feeding her brother	Maize and okro soup	
5:10	FS	SB	Hand feeding her brother	Maize meal and okro soup	
5:15	FS	SB	Feeding her brother	Maize meal and okro soup	
5:20	SB		Continues to eat alone	Maize and okro soup	
5:25	SB		Eating alone	Maize meal and okro soup	
5:30	SB		Eating alone from the plate		
5:40	SB		Drop the cup of water		
5:50	FS	SB	Washing her brothers body		
6:00	FS	SB	Carry her brother and keep him on the mat		
6:05	SB		Start playing		

TIME	ACTOR INITIALS	RECIPIENT INITIALS	WHAT HAPPENED	FOOD	Reactivity

TIME	ACTOR INITIALS	RECIPIENT INITIALS	WHAT HAPPENED	FOOD	Reactivity

PARTICIPANT ID: __ _______

Primary Questions (Complete after you complete data collection):

From what you have seen observing THIS HOUSEHOLD, please answer the following questions in detail:

Question	Write your detailed response here
1. In this household, what would be the best way(s) to give nutritional supplements such as an MNP to young children to ensure compliance?<br><br>Why?	The best way and time to give the MNP should be in the morning because that is when children are fed with semi solid food.<br><br>Because kunu (pap) is usually prepared for young children in the morning , all other foods are usually solid foods
2. In this household, who is favored during meal preparation or feeding?	Nobody is favored during feeding because all the young children gather to eat except in the morning when every child has a separate cup for kunu.

How is this favoritism expressed?	All the children are served the same quantity of either kunu or solid foods that they all ate together
3. How could we promote the MNP supplement for this family that would attract their attention?<br><br>Who would they listen to?	MNP can be promoted to this family through the mother because she is the person in charge of meal preparation and feeding.<br><br>They would listen to the mother because she is the one that is responsible for their upbringing and also their welfare.
4. In this household, what would be the best ways to promote the MNP but limit how much it would be shared?<br><br>Why?	The best way would be through the mother and the possibility that the MNP would be shared is limited.<br><br>Because every child has a separate cup for kunu in the morning that makes it easy for the mother to quickly pour the MNP without the other children notice.
5. What other issues and concerns did you see with this family that are important for us to consider with regard to introduction of an MNP?<br><br>Why?	The general hygiene of this household is very poor , the children do not wash their hands before eating and the mother leaves the food items open to flies.<br>The young child is not given necessary care and attention. The only time he gets the mother's attention is when it is time to mixed MNP and kunu.
6. Based on your experience observing this child today, do you believe reactivity negatively impacted your ability to see the food-related	No, the mother and other children are used to me already. They are more relaxed.<br><br>There was no reactivity.

behaviors of the child?<br><br>Please explain your answer in detail.	
